# PVA/PANI-DBSA Nanomesh Tactile Sensor for Force Feedback

**DOI:** 10.3390/polym16111449

**Published:** 2024-05-21

**Authors:** Boyi Wang, Rong Du, Yi Liu, Han Song

**Affiliations:** 1 School of Mechanical and Electrical Engineering, Wuhan University of Technology, Wuhan 430070, China; 2 Hubei Key Laboratory of Digital Manufacturing, Wuhan University of Technology, Wuhan 430070, China; 3 Shaoxing Institute for Advanced Research, Wuhan University of Technology, Shaoxing 312300, China

**Keywords:** piezoresistive, tactile sensors, electrospinning, nanomesh, PANI-DBSA

## Abstract

Touch serves as an important medium for human–environment interaction. The piezoresistive tactile sensor has attracted much attention due to its convenient technology, simple principle, and convenient signal acquisition and analysis. In this paper, conductive beads-on-string polyvinyl alcohol (PVA)/polyaniline doped with dodecyl benzene sulfonic acid (PANI-DBSA) nanofibers were fabricated via the electrospinning technique. Due to the special nanostructure of PVA-coated PANI-DBSA, the tactile sensor presented a wide measuring range of 12 Pa–121 kPa and appreciable sensitivity of 8.576 kPa^−1^ at 12 Pa~484 Pa. In addition, the response time and recovery time of the sensor were approximately 500 ms, demonstrating promising prospects in the field of tactile sensing for active upper limb prostheses.

## 1. Introduction

Upper limb amputees have many limitations in their daily activities, and current artificial prostheses are unable to resolve these limitations completely [1,2]. Most commercial prostheses lack force or tactile feedback, seriously hindering the hand’s ability to grasp [3]. Touch plays a critical role in our ability to perceive and manipulate the world and is an essential prerequisite for fine manipulation [4,5]. In a more comprehensive definition, touch includes the perception of pressure, vibration, slip, and texture. In a more specific definition, it is the sensation of force [6]. Most users (98%) desired to feel the force applied by the prosthesis during grasping [7]. As a crucial component of haptic interaction, force feedback has become an indispensable function for haptic interaction. Therefore, pressure measurement is the most important aspect of tactile sensing [8,9,10]. Over the last decade, many pressure sensors have been developed based on different sensing mechanisms such as piezoresistive [11,12], piezocapacitive [13,14], piezoelectric [15,16], and triboelectric [17,18]. However, piezoelectric sensors are unable to detect constant pressure, and triboelectric pressure sensors suffer from a lack of stability. Compared to other types, piezoresistive sensors offer superior stability and constant pressure detection [19,20] due to their simple structure, ease of preparation, cost effectiveness, and straightforward signal acquisition and analysis. Thus, improving the performance of piezoresistive pressure sensors has become a critical topic for researchers.

In mass production, electrospinning is a cost-effective and highly efficient technique for continuous nanofiber fabrication [21]. However, there are some challenges that need to be solved when using the electrospinning technology to prepare piezoresistive pressure sensors. Most suitable polymers for electrospinning are non-conductive, which indicates that the electrical signal cannot be generated and transmitted. Thus, mixing electrospinning solutions with conductive fillers (such as carbon nanotubes [22,23,24], graphene [25], or metal nanowire [26]) is one of the best methods to modify the non-conductive polymer nanofibers to conductors. As a conductive polymer material, polyaniline (PANI) has the advantages of low density, high conductivity, and good plasticity [27]. By combining PANI with other polymer materials, composite nanofibers with good conductivity can be obtained by electrospinning technology. Jin et al. [28] prepared orientated thermoplastic polyurethane elastomer rubber nanofiber membranes (TPU-O NMs) via conjugated electrospinning, and PANI was in situ polymerized on TPU-O NMs as flexible electrodes. The assembled piezocapacitive pressure sensor showed exceptional performance.

Additionally, low sensitivity to small pressure is the most common problem for piezoresistive pressure sensors [29]. To improve the sensitivity of the sensor for small pressure detection, surface microstructures of the pressure sensors (i.e., domes [30,31], pyramids [32,33], and columnar [34] structures) can be designed. In recent times, this method has been extensively applied to improve the sensitivity, response speed, and stability of piezoresistive pressure sensors [35,36].

With the advancements in the photolithography technique, an increasing number of researchers are focusing on the utilization of this technique to design templates for acquiring surface microstructures. Park et al. [31] used multi-walled carbon nanotube/polydimethylsiloxane composite film as the sensor’s active material and flat indium tin oxide film as the contact electrode to fabricated flexible piezoresistive pressure sensors with engineered microstructure geometries. The sensitivity of the microdome and micropyramid was 18.3 kPa^−1^ and 12.6 kPa^−1^, respectively, when the pressure was less than 1 kPa. But the measuring range was less than 26 kPa. Peng et al. [37] sprayed a thin layer of conductive carbon nanofibers on textured PDMS film and fabricated an ultra-sensitive flexible piezoresistive pressure sensor based on a micro-semicylinder surface structure. Although the sensor possessed a high sensitivity of −3.6 kPa^−1^, its measuring range was less than 2 kPa. The surface microstructure confers excellent sensitivity on the sensor, but the measuring range of the sensor is limited due to the limited bearing capacity of the surface microstructure. Furthermore, photolithography is expensive, time-consuming, and intricate, which renders such sensors unsuitable for use in force feedback systems in prosthetic limbs. Therefore, an alternative, low-cost approach is to use readily available materials (such as sandpaper [38], leaves [39], or silk [40]) as a mold due to their unique surface microarchitecture. Pang et al. [41] designed a flexible piezoresistive pressure sensor with a spinous microstructure using sandpaper as a template. The sensitivity was as high as 25.1 kPa^−1^ in a linearity range of 0~2.6 kPa. However, the lack of homogeneity in the microstructure of the sensor results in limitations in its repeatability, lifetime, and measurement range. To increase the measuring range, Liu et al. [42] obtained silk fibroin/graphene nanomembranes by double-needle electrospinning and carried out encapsulation by polydimethylsiloxane to prepare a pressure sensor. Although the maximum working pressure was improved to 200 kPa, the sensitivity of the sensor was only 7.7 Pa^−1^. Therefore, there is a trade-off between the detection range, sensitivity, and fabrication cost of the piezoresistive pressure sensor.

In this research, we report a conductive nanomesh comprising PVA/PANI-DBSA nanofibers fabricated by electrospinning. A PVA/PANI-DBSA tactile sensor with good sensitivity and responsiveness was obtained by PDMS packaging. As a conductive polymer material, PANI-DBSA has the advantages of high conductivity and good plasticity. Therefore, the PVA/PANI-DBSA nanofibers produced through electrospinning exhibit excellent elasticity and conductivity. During the process of electrospinning, PANI-DBSA nanoparticles were dispersed in PVA nanofibers. However, this dispersion was not always homogeneous, and some PANI-DBSA nanoparticles gathered in nanofibers, which resulted in the PANI-DBSA-enriched regions on the nanofibers exhibiting greater conductivity than other regions. When pressure is applied to the nanomesh, the conductive nanofibers make contact, creating additional conductive paths and ultimately reducing the sensor’s overall resistance. Therefore, the PVA/PANI-DBSA tactile sensor possesses immense potential for application in the area of force feedback for active prostheses.

## 2. Materials and Methods

### 2.1. Materials

Polyvinyl alcohol (PVA, Mw~67000 g/mol) was purchased from Sigma-Aldrich (Shanghai, China), and polyaniline doped with dodecyl benzene sulfonic acid (PANI-DBSA) was bought from Shanghai Macklin Biochemical Technology. All chemicals were analytical reagent and were used without alteration.

### 2.2. Fabrication

#### 2.2.1. Configuration of Electrospinning Solution

**Figure 1:**
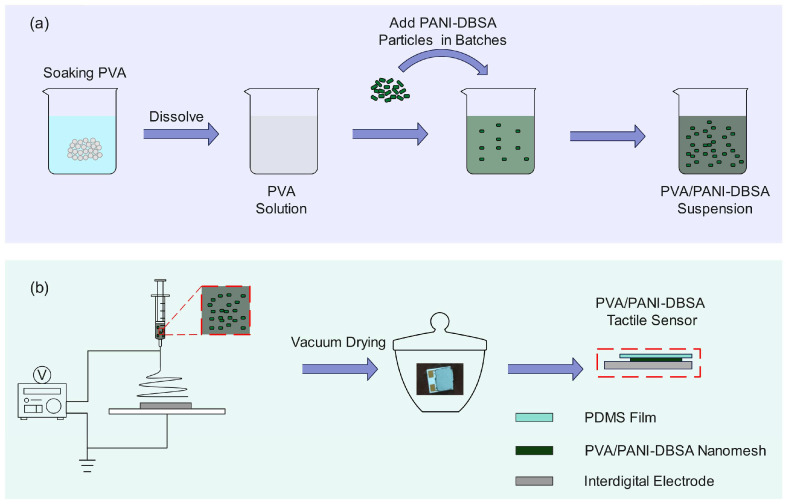
(**a**) Preparation of PVA/PANI-DBSA solution. (**b**) schematic diagram of preparing PVA/PANI-DBSA nanofiber membrane by electrospinning.

The preparation process of electrospinning precursor solution is shown in Figure 1a. Initially, 2 g PVA was submerged in 10 mL of deionized water for 3 h and was then stirred using a magnetic stirrer at 70 °C for 24 h until a homogeneous solution (transparent color) was attained. The solution was then left for 12 h undisturbed in order to release any trapped bubbles. Secondly, 1.8 g of PANI-DBSA powder was added to the aforementioned PVA solution in batches and continuously stirred at 65 °C throughout the addition process to produce a consistent PVA/PANI-DBSA suspension (deep green).

#### 2.2.2. Fabrication of PVA/PANI-DBSA Tactile Sensor

Electrospinning is a unique technique that allows the production of single or multiple layers of continuous fibers with diameters ranging from submicron to nanometer. Therefore, polymer nanofibers can achieve a finer diameter compared to traditional technology, while the nanofiber web produced by using nanofibers has a higher specific surface area. In the electrospinning process, the inherent properties of the polymer solution and the operating conditions determine the shape and diameter of the fiber. The type of polymer, solvent concentration, and conductivity are key factors in determining the morphology and diameter of the fiber, but voltage, extrusion speed, tip-to-collector distance, temperature, and humidity also affect the diameter of the fibers.

To reduce the influence of humidity and temperature on the diameter and morphology of fibers, the electrospinning process was carried out at a constant temperature and humidity laboratory at 25 °C and 45%RH. The sensor preparation process is shown in Figure 1b: 1 mL of the suspension prepared in the preceding step was moved into a plastic syringe, and the electrospinning process took place for 10 in a confined environment. The parameters of the process were set as follows: voltage: 14 kV, tip-to-collector distance: 7.5 cm, solution flow rate: 0.15 mL/h, and inside diameter of the needle: 0.6 mm. The nanofibers were collected on top of the interdigitated electrodes placed on the ground collector and by vacuum drying 12 h prior to use. Then, a polydimethylsiloxane (PDMS) film was placed on the PVA/PANI-DBSA nanomesh to provide encapsulation for PVA/PANI-DBSA tactile sensor.

### 2.3. Characterization

The surface morphology of PVA/PANI-DBSA nanofiber membranes was analyzed by a scanning electron microscope (SEM) (Zeiss Ultra Plus, Carl Zeiss, Oberkochen, Germany). In addition, a transmission electron microscope (TEM) (Talos F200S, Thermo Fisher, Waltham, MA, USA) was used to analyze the distribution of PANI-DBSA particles in PVA nanofibers, while scanning transmission electron microscopy–energy dispersive X-ray spectroscopy (STEM-EDS) was used to investigate the elemental composition. The diameter distribution of nanofibers was analyzed using the software ImageJ (v1.8.0.345, National Institutes of Health, Bethesda, MD, USA) by SEM images.

### 2.4. Experimental Design

**Figure 2:**
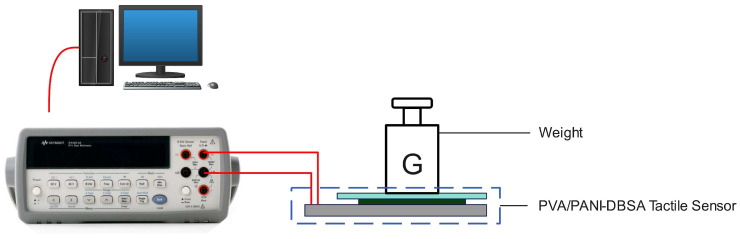
Pressure test device for PVA/PANI-DBSA tactile sensors.

The experimental diagram is shown in Figure 2. The performance evaluation of the PVA/PANI-DBSA tactile sensor was performed by loading various standard weights (0.05 g, 0.1 g, 0.5 g, 1 g, 2 g, 5 g, 10 g, 20 g, 50 g, 100 g, 200 g, and 500 g). The load applied was converted to pressure by dividing it by the upper surface area of the sensor, which is 40.5 mm^2^. Furthermore, PDMS film was employed to isolate the weights with nanofibers. A digital multi-meter (34401A, Agilent Technologies, Santa Clara, CA, USA) was used to collect signals of resistance of the fabricated sensor at 26 °C and 60 ± 5% humidity. Finally, the collected signal was recorded on a computer using LabVIEW (2021 SP1) software.

## 3. Results and Discussion

### 3.1. Structural Characterization

**Figure 3:**
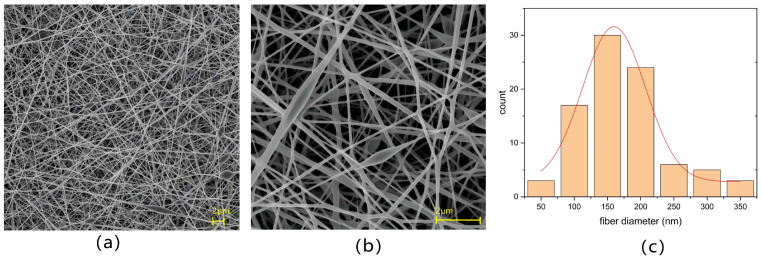
SEM images of PVA/PANI-DBSA nanofibers at different magnifications: (**a**) 2000× and (**b**) 8000×. (**c**) The nanofibers’ diameter distribution analyzed according to (**a**).

**Figure 4:**
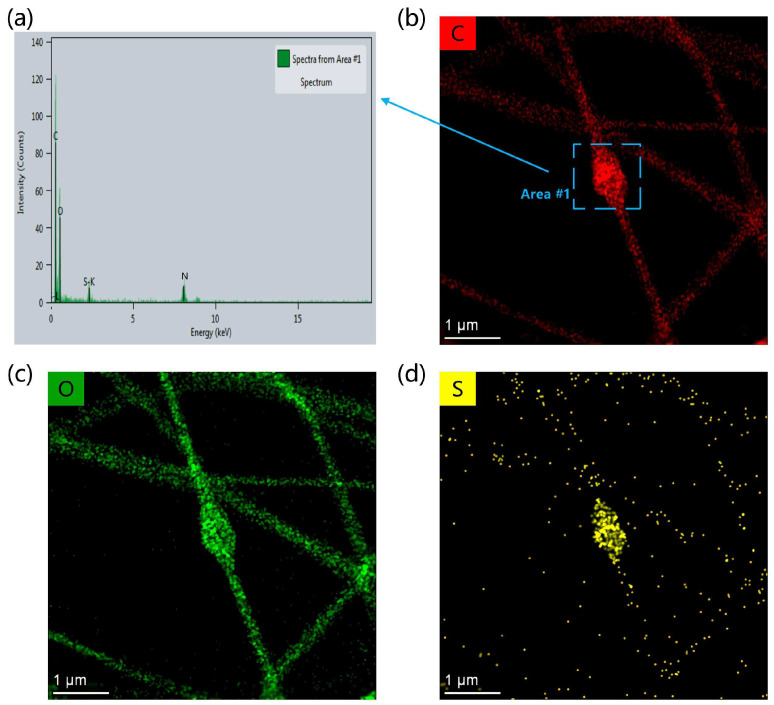
(**a**) The energy spectrum of Area #1. STEM-EDS maps of C (**b**), O (**c**), and S (**d**) of PVA/PANI-DBSA nanofibers.

In order to determine the morphology of the nanomesh, the SEM images of the PVA/PANI-DBSA nanomesh are presented in Figure 3a,b. The beads-on-string PVA/PANI-DBSA nanofibers were smooth and formed a continuous fiber network structure. Furthermore, there were pores with different sizes and many joints among nanofibers. Figure 3c presents the diameter distribution of nanofibers analyzed according to Figure 3a by the software ImageJ. It can be observed that the diameter of nanofibers was predominantly distributed within the range of 100–200 nm, which gave the PVA/PANI-DBSA nanomesh a good degree of elasticity. At the same time, the thinner nanofibers can obtain a lower detection limit and improve the sensitivity of the sensor at low pressure. Additionally, as shown in Figure 3a,b, the nanofibers had a large number of beads, most of which were larger than 2 μm in length and width. The presence of beads was primarily attributed to the enrichment of PANI-DBSA nanoparticles. This agglomeration phenomenon can be attributed to the direct addition of PANI-DBSA nanoparticles to the PVA solution without using organic solvents to dissolve, and another possible reason is insufficient stirring speed and duration. To further illustrate the influence of the existence and distribution of PANI-DBSA nanoparticles on the structure and elemental composition of nanofibers, a beads-on-string nanofiber was characterized by STEM-EDS. Figure 4a shows the energy spectrum of Area #1. In Figure 4b–d, EDS mapping analysis reveal the coexistence of C, O, and S in the sample. In addition, the distribution of S demonstrates the uneven distribution of PANI-DBSA nanoparticles in fibers. Therefore, the composite structure enhanced nanofiber conductivity and elasticity while ensuring a stable conductive network within the multilayer network, even during deformation.

### 3.2. Sensing Performance of PVA/PANI-DBSA Tactile Sensor

**Figure 5:**
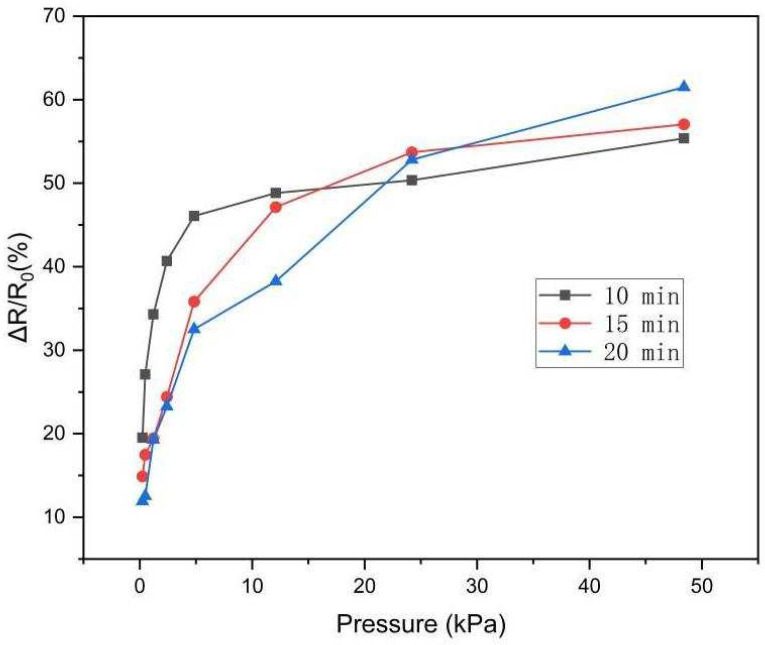
A comparison of the relative resistance changes of three different electrospinning durations.

Figure 5 shows the curve of the relative resistance change of the sensor under pressure for different electrospinning durations. When the pressure was below 12.1 kPa, the relative resistance of the sensor with smaller thickness (10 min) changed more than that of the other two. On the other hand, when the pressure exceeded 24.2 kPa, the relative resistance change of the thicker sensor (20 min) was higher than that of the others. The reason for this phenomenon is that the brief duration makes the PVA/PANI network relatively thin. Consequently, even minor pressure can induce a considerable deformation. In response to the same external pressure, the deformation of thick film accounts for a small proportion of its own maximum deformation. Therefore, when the pressure is less than 12.1 kPa, the relative resistance of the thin network under the same pressure changes significantly. However, when the pressure exceeds 24.2 kPa, the deformation of the thin network tends to be saturated, while the deformable space of the thick network is larger, resulting in a significant change in relative resistance. Therefore, the electrospinning time of the sensor used for subsequent performance tests was 10 min.

**Figure 6:**
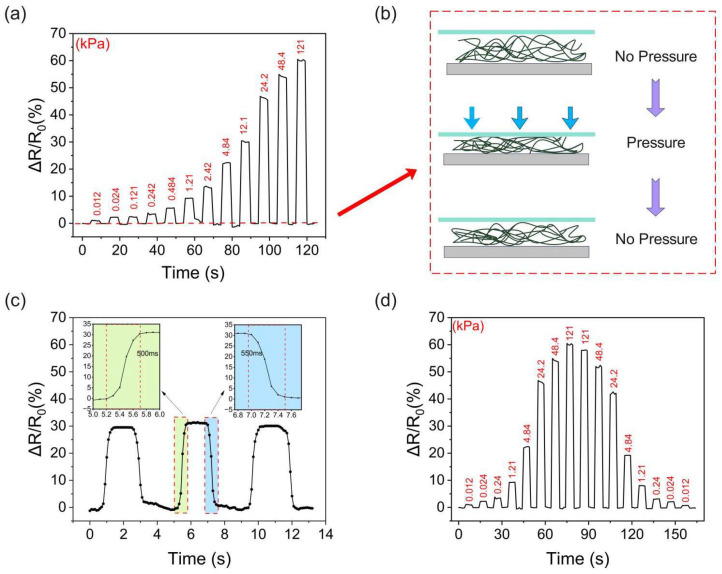
(**a**) Relative resistance of the sensor when different pressures are applied. (**b**) The reason for the initial value of relative resistance fluctuates during compression. (**c**) The response and recovery time of the sensor. (**d**) The variations in the sensor’s relative resistance during the compression and release processes.

The performance of the pressure sensor can be assessed by its response time, sensitivity, and hysteresis. In order to unify the graph data, we chose to use the relative resistance change as a parameter. During the measurement, the initial resistance of the sensor was 40 MΩ. The relative resistance change as a function of the applied pressure is shown in Figure 6a. The nanomesh underwent deformation as the pressure was applied on the surface of the sensor, which led to a change in the internal conductive path of the nanomesh. Therefore, the overall resistance of the sensor changed with the various applied pressure. Notably, the sensor can perceive as little as 12.1 Pa of pressure. In addition, with the gradual increase in pressure, the change in the relative resistance of the sensor becomes faint, which suggests that the sensor is not sensitive to high loads due to the shrinking of the deformable space of nanofiber networks. As shown in Figure 6b, the plastic deformation of the few PVA/PANI-DBSA nanofibers occurred during compression, which resulted in the nanofibers being difficult to restore to their original form after the applied pressure release. Therefore, the initial relative resistance of the sensor was slightly unstable after few cycles, and the variation range was ±2%. Nevertheless, as compression increased, the initial value of the sensor resistance tended to stabilize. This is evident in the curve of the resistance response and applied pressure of the PVA/PANI-DBSA tactile sensor at 4.84 kPa shown in Figure 6c. The sensor swiftly reacted to pressure, and the response and recovery times of the sensor equated to 90% of the period when the sensor achieved a stable state of relative resistance, which was about 500 ms. Moreover, hysteresis, a vital metric for gauging sensor performance, affects the precision of sensor measurement. Figure 6d illustrates the variations in the sensor’s relative resistance during the compression and release processes, with the calculation showing that the hysteresis error of the sensor was less than 7%. The hysteresis error ($e_{H}$) is calculated as follows:

(1) $$e_{H}=\frac{∆H_{max}}{Y_{FS}}\times 100\%$$

where $∆H_{max}$ is the maximum difference of the compression and release processes, and $Y_{FS}$ is the full-scale output value. The output value of the sensor indicated that the average value of $∆H_{max}$ was 4.153 at 24.2 kPa, while the average value of the full-scale output of the sensor was 60.125. Therefore, the hysteresis error was calculated to be 6.9% according to Formula (1).

**Figure 7:**
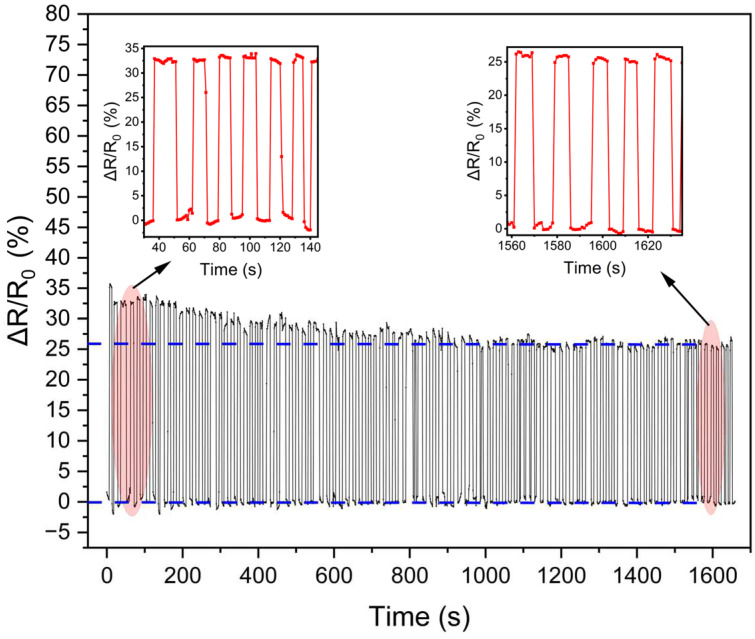
Cycling stability of the as-prepared sensor over 100 cycles under 4.84 kPa.

As illustrated in Figure 7, the prepared sensor was subjected to 100 cycles at 4.84 kPa to assess its repeatability. During the initial cycle, it can be observed that the peak of the sensor’s relative resistance change gradually decreased and tended to stabilize, which can be attributed to the plastic deformation of the nanofibers. In the initial cycles, the number of nanofibers involved in deformation was greater than that in the stable cycles. Consequently, during the initial cycle, the relative resistance of the sensor underwent a more pronounced change. However, as the cycle progressed, the number of nanofibers undergoing plastic deformation gradually decreased, and eventually, the peak of the relative resistance change of the sensor was essentially the same.

In the case of piezoresistive pressure sensors, sensitivity is defined as:

(2) $$\Delta R=R_{0}\;-\;R$$

(3) $$S=\frac{\delta \left(\frac{\Delta R}{R_{0}}\right)}{\delta P}$$

where $R_{0}$ is the initial resistance of the sensor under no pressure. ∆R is the relative change in resistance when the sensor is subjected to pressure. P is applied pressure.

**Figure 8:**
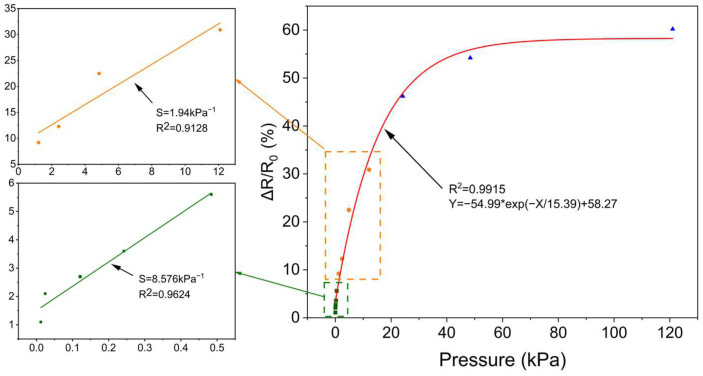
The relative resistance of the sensor changes with applied pressure.

Due to the special structure of dispersed PANI-DBSA particles wrapped in PVA nanofibers, the sensor not only has a fast response time and a large detection range but also good sensitivity. As shown in Figure 8, to evaluate the sensing performance of the sensor, the curve of the voltage response versus applied pressure was plotted, revealing a linear range of 0.012 kPa to12.1 kPa. According to Formula (3), the slope of the fitted line is the sensitivity of the sensor. When the pressure is less than 0.484 kPa, the sensor exhibits a sensitivity of approximately 8.576 kPa^−1^ and about 1.94 kPa^−1^ at pressures ranging from 0.484 kPa to 12.1 kPa. Due to the variation in the compression space of the sensor, the sensitivity is divided into two parts. The data show that the sensitivity of the sensor has a non-constant trend, which gradually decreases with the increase in pressure. When the pressure falls below 12.1 kPa, the reduction in sensitivity is negligible. Therefore, the range between 0.012 kPa and 12.1 kPa was split into two linear intervals. However, sensitivity significantly decreases in the pressure range between 12.1 kPa and 121 kPa. The observed discrepancy in sensitivity can be attributed to variations in the deformable space of the nanofiber network. When subjected to pressure, the nanofiber network undergoes deformation, resulting in contact between the nanofibers, particularly between the beads and other parts. As the deformation space change tends to saturate at high pressures, the sensitivity of the sensors decreases accordingly. Therefore, the resistance change at the low-pressure range (12 Pa–0.484 kPa) has higher sensitivity.

### 3.3. Working Mechanism of PVA/PANI-DBSA Tactile Sensor

Piezoresistive pressure sensors with a surface microstructure have the same phenomenon of a gradual decline in sensitivity and change in initial resistance value. At low pressure, it is concentrated near the contact tip due to the small contact area, which leads to higher mechanical deformation and rapid changes in the contact area. However, with the further increase in applied pressure, deformation of the microstructure tends to be saturated, and due to the large cross-sectional area of the compressed structure, the additional pressure will only cause a slower change in the contact area. Therefore, the gradual decline in sensitivity can be attributed to a gradual reduction in the deformable volume of the sensor.

Although the sensitivity change of the piezoresistive sensor with a surface microstructure is identical to that of a nanofiber piezoresistive sensor, the working mechanisms are distinct. The change in resistance of the former sensor is primarily attributed to the alteration in effective contact area resulting from surface microstructure, which induces a more pronounced variation in contact resistance. The latter is primarily attributable to the deformation of the beads-on-string nanofibers, which facilitates the formation of a conductive path between them, thereby reducing sensor resistance.

**Figure 9:**
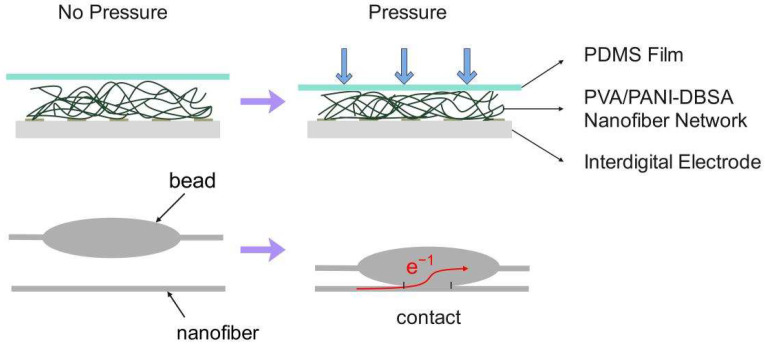
Schematic illustration of the main structure change of the sensor during the compression process.

Figure 9 presents a schematic illustration of the structural change that occurs in the sensor during the compression process. The total resistance (R) of the sensor consists of the resistance ($R_{e}$) of the electrode, the volume resistance ($R_{v}$) of the PVA/PANI network, and the contact resistance ($R_{c}$) between the PVA/PANI network and the electrode. The calculation formula is as follows:

(4) $$R=R_{e}+R_{v}+R_{c}$$

The value of Re is fixed and related to the resistance value of the electrode. In the case where the nanofiber network and the electrode are not in close contact, the contact area between the two will increase with the increase in external force, which causes a change in the contact resistance ($R_{c}$) between the two interfaces. The results of the repeatability test demonstrate that the sensor resistance undergoes greater fluctuations during the initial cycle than during the stable cycle. This phenomenon can be attributed to two primary factors. Firstly, the sensor nanofiber network exhibits reduced contact with the electrode. Secondly, there is a greater number of nanofibers without plastic deformation. Of course, there are also contact resistance changes in the contact of the nanofiber, and we incorporate the contact resistance changes between the nanofibers into the volume resistance ($R_{v}$). The pressure of the sensor changes the contact state of the conductive nanofibers, and the contact resistance of these nanofibers also changes while the new conductive path is generated, which leads to the regular change in $R_{v}$ with pressure.

The PVA/PANI pressure sensor exhibited a high sensitivity of up to 8.576 kPa^−1^ when the pressure was less than 0.484 kPa, which is significantly higher than that of the high-pressure region (>12.1 kPa). It is evident that when subjected to a small pressure of less than 0.484 kPa, the nanofiber is prone to deformation due to its small diameter. Concurrently, the deformable space of the nanofiber network is considerable, facilitating the formation of a conductive path through the contact of numerous nanofibers, which results in a rapid reduction in $R_{v}$ and a significant change in sensor resistance. In the second stage of nanofiber deformation (0.484–12.1 kPa), the average sensitivity was 1.94 kPa^−1^. At this point, higher pressures are required for further deformation. In the third stage (>12.1 kPa), the sensitivity of the sensor declined rapidly, and the deformation space of the nanonetwork tends to be saturated.

## 4. Application

The human hand is a powerful tool for perceiving and manipulating the environment, as well as a very complex means of physical and social interaction. For unilateral patients, the prosthesis is primarily an aid to the sound limb, while for subjects with bilateral limb loss, the prosthesis is the primary way to interact with the environment. Most patients desired to feel the force feedback of the prosthesis during grasping. However, the lack of force feedback in current commercial active prostheses limits the improvement in the grasping ability of patients, thus limiting the recovery of the function of the patient’s arm. Additionally, the manufacturing of commercial tactile sensors is complex, making their cost high and inhibiting their practical application.

**Figure 10:**
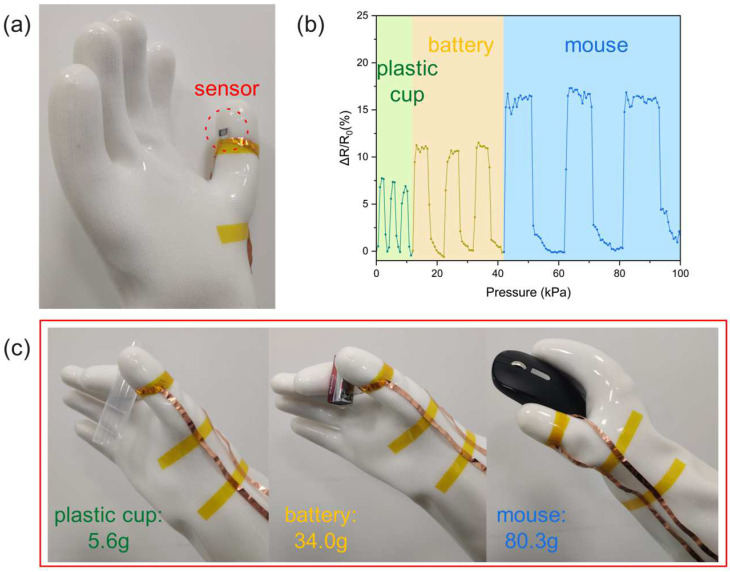
(**a**) The sensor attached to silicone-insulated gloves. (**b**) Variation in resistance over time with hand grasping and releasing. (**c**) The weight of different items.

The PVA/PANI-DBSA tactile sensor prepared in this paper has advantages in force feedback. Data are provided to the patient for force feedback by attaching sensors to the prosthesis to monitor pressure in real time as the prosthesis grasps. In order to simulate tactile sensing applications, the sensor was attached to silicone-insulated gloves. Figure 10a demonstrates the use of gloves to grasp and release various items, while Figure 10b depicts the resulting graph. In summary, due to the special structure of dispersed PANI-DBSA particles wrapped in PVA nanofibers, the PVA/PANI-DBSA tactile sensor has a good sensitivity and measuring range. In addition, the tactile simulation test demonstrates the feasibility of using low-cost pressure sensors based on PVA/PANI-DBSA nanomesh and fabricated by the electrospinning techniques for force feedback for prosthetic limbs.

## 5. Conclusions

In this paper, we first proposed a new structure of dispersed PANI-DBSA particles wrapped in PVA nanofibers by electrospinning technology for the preparation of high-sensitivity pressure sensors. The special structure exhibited exceptional performance. The sensitivities of the sensors prepared in the pressure ranges 0.012 kPa to 0.484 kPa and 0.484 kPa to 12.1 kPa were 8.67 kPa^−1^ and 1.94 kPa^−1^, respectively. In addition, the sensor had a response and recovery time of about 500 ms and a hysteresis error of less than 7%. Moreover, the preparation of the sensor is simple and low-cost. To simulate force feedback, an object-grabbing experiment was carried out by attaching sensors to insulated gloves, and the results show that the sensor has excellent dynamic pressure response. Therefore, the PVA/PANI-DBSA tactile sensor prepared by electrospinning technology has broad prospects in the field of force feedback for active upper limb prostheses.

## Data Availability

Data are contained within the article.
